# Bacteroides-dominant gut microbiome of late infancy is associated with enhanced neurodevelopment

**DOI:** 10.1080/19490976.2021.1930875

**Published:** 2021-06-16

**Authors:** Sukhpreet K. Tamana, Hein M. Tun, Theodore Konya, Radha S. Chari, Catherine J. Field, David S. Guttman, Allan B. Becker, Theo J. Moraes, Stuart E. Turvey, Padmaja Subbarao, Malcolm R. Sears, Jacqueline Pei, James A. Scott, Piush J. Mandhane, Anita L. Kozyrskyj

**Affiliations:** a Department of Pediatrics, University of Alberta, Edmonton, AB, Canada; b HKU-Pasteur Research Pole, School of Public Health, Li Ka Shing Faculty of Medicine, University of Hong Kong, Hong Kong SAR, China; c Dalla Lana School of Public Health, University of Toronto, Toronto, ON, Canada; d Department of Obstetrics and Gynecology, University of Alberta, Edmonton, AB, Canada; e Department of Agricultural, Food & Nutritional Science, University of Alberta, Edmonton, AB, Canada; f Centre for the Analysis of Genome Evolution and Function, University of Toronto, Toronto, ON, Canada; g Department of Pediatrics & Child Health, Children’s Hospital Research Institute of Manitoba, University of Manitoba, Winnipeg, MB, Canada; h Department of Pediatrics, Hospital for Sick Children, University of Toronto, Toronto, ON, Canada; i Department of Pediatrics, Child & Family Research Institute, BC Children’s Hospital, University of British Columbia, Vancouver, BC, Canada; j Department of Medicine, McMaster University, Hamilton, ON, Canada; k Department of Educational Psychology, University of Alberta, Edmonton, AB, Canada

**Keywords:** Infant, gut microbiota, neurodevelopment, cognition, bacteroidetes, early colonization, birth cohort

## Abstract

Dysbiosis of gut microbiota has been retrospectively linked to autism spectrum disorders but the temporal association between gut microbiota and early neurodevelopment in healthy infants is largely unknown. We undertook this study to determine associations between gut microbiota at two critical periods during infancy and neurodevelopment in a general population birth cohort.

Here, we analyzed data from 405 infants (199 females) from the CHILD (Canadian Healthy Infant Longitudinal Development) Cohort Study. Neurodevelopmental outcomes were objectively assessed using the Bayley Scale of Infant Development (BSID-III) at 1 and 2 years of age. Microbiota profiling with 16S rRNA gene sequencing was conducted on fecal samples obtained at a mean age of 4 and 12 months.

Using clustering methods, we identified three groups of infants based on relative abundance of gut microbiota at 12 months: *Proteobacteria*-dominant cluster (22.4% higher abundance at 12 months), *Firmicutes*-dominant cluster (46.0% higher abundance at 12 months) and Bacteroidetes-dominant cluster (31.6% higher abundance at 12 months). Relative to the *Proteobacteria*-dominant cluster, the *Bacteroidetes*-dominant cluster was associated with higher scores for cognitive (4.8 points; FDRp = .02), language (4.2 points; FDRp≤0.001), and motor (3.1 points; FDRp = .03) development at age 2 in models adjusted for covariates. When stratified by sex, only male infants with a *Bacteroidetes*-dominant microbiota had more favorable cognitive (5.9 points, FDRp = .06) and language (7.9 points; FDRp≤0.001) development. Genus *Bacteroides* abundance in gut microbiota was positively correlated with cognitive and language scores at age 2. Fully adjusted linear mixed model analysis revealed a positive association between *Bacteroidetes*-dominant cluster and change in cognitive and language performance from 1 to 2 years, predominantly among males. No associations were evident between 4-month microbiota clusters and BSID-II scores. Noteworthy is that enhanced sphingolipid synthesis and metabolism, and antagonism or competition between *Bacteroides* and *Streptococcus* were characteristic of a *Bacteroidetes*-dominant gut microbiota.

This study found strong evidence of positive associations between Bacteroidetes gut microbiota in late infancy and subsequent neurodevelopment, most prominently among males but not females.

## Introduction

Neurodevelopmental disorders (e.g. autism, attention-deficit/hyperactivity disorder, learning disabilities) manifest early in development and result in lifelong deficits in cognitive, social, emotional, academic, and adaptive functioning.^1^ The number of children affected by a developmental disorder now represents 13.4% of children aged 6 to 17^2–4^ and 20.1% of children aged 1 to 7^5^ years old worldwide. Environmental factors are the primary drivers of neurodevelopment in very early childhood^6^ and genetic variations in brain signaling pathways can be modified by early life environments.^7^ The gut microbiome is altered in children with well-defined phenotypes of developmental delay such as autism,^8–11^ or risk factors for developmental delay, such as preterm birth.^12,13^ It is becoming increasingly clear that gut microbiota influences brain function and behavior through signaling pathways of the microbiome-gut-brain axis.^14^

Starting with colonization by facultative anaerobes, such as the lactic acid bacteria, then followed by an expansion of strict anaerobes within the Firmicutes (e.g. genus *Ruminococcus, Veillonella*) and Bacteroidetes (e.g. genus *Bacteroides*) phyla,^15,16^ the trajectory of the gut microbiome across infancy coincides with key neurodevelopmental periods.^17^ Peak abundance of lactic acid bacteria and bifidobacteria in the infant gut post birth coincides with the time period for aspects of neuronal development related to synaptogenesis and myelination.^15,18^ Since microbial signals are hypothesized to be critical for establishing the gut-brain axis,^17^ early life exposures that shape gut microbiota such as cesarean delivery,^19^ formula-feeding,^20^ and antibiotic treatment,^21^ are not inconsequential. Indeed, several first-colonizing microbiota, as well as members of the Bacteroidetes, are depleted for several weeks after birth by cesarean section,^15,21,22^ which is a risk factor for developmental delay.^23^

Whereas changes in the infant gut microbiome coincide with a critical period in early brain development, little is known about their relation to developmental and behavior outcomes. In a cross-sectional study of 77 toddlers at age 2, Christian et al.^24^ found greater gut microbial diversity and abundance of taxa in the Bacteroidetes phylum (i.e. *Parabacteroides*) to be associated with infant temperament based on parent report. Longitudinal studies have shown other taxa in the Bacteroidetes phylum (i.e. *Prevotella*), when depleted in late infancy, to be associated with internalizing behaviors at age 2^25^ but found few associations between *Bacteroides*-dominant microbiota at age 2 months and temperament in 6-month-old infants.^26^ Similarly, the Carlson et al.^27^ cohort study of 89 infants found enrichment of gut microbiota with genus *Bacteroides* at 12 months to be associated increased cognitive development on the Mullen scale at 2-years old. On the other hand, Sordillo et al. 2019^28^ reported that a *Bacteroides*-dominant gut microbiota at 3–6 months was associated with increased odds for delayed fine motor skills in 309 preschool children assessed by the Ages and Stages Questionnaire. Further, these small-scale studies did not all report on gut microbial function or sex differences in neurodevelopmental outcomes. Sex differences in brain development are well established^29,30^ and likely account for sex-specific skill acquisition in domains like verbal communication.^31,32^ Finally, there is very little information regarding the function of infant gut microbiota underlying healthy neurodevelopment.

Using data from the large population-based CHILD (Canadian Healthy Infant Longitudinal Development) Cohort Study, we examined temporal associations between gut microbiota composition and function during infancy and neurodevelopmental outcomes at 1 and 2 years of age. We aimed to identify microbial clusters and their relation to three objectively-assessed neurodevelopmental domains (cognitive, language, motor). We hypothesized that genus *Bacteroides* would be associated with enhanced neurodevelopmental scores in a sex-specific manner.

## Results

### Participant characteristics

Among the 405 study infants with complete data, 50.9% were males, 53.8% had an older sibling, and 26.4% were born by cesarean delivery. At 6 months of age, a large proportion (73.6%) of infants were partially or exclusively breastfed. Other than siblingship, these characteristics did not differ significantly from infants without fecal samples that were not available for study (Table S1). Study infants were from higher income families and their mothers were more highly educated than infants excluded from analysis. Among infants with neurodevelopmental outcomes assessed using the Bayley Scale of Infant Development (BSID-III) at 2 years of age, the mean and standard deviation (SD) for the cognitive composite was 105.6 (SD *= *14.2), language composite was 100.7 (SD *= *11.9), and motor composite was 99.1 (SD *= *9.6), shown in Table 1. Females had a higher mean cognitive score (Mean = 108.4 vs 102.9, p ≤ .0001), and mean language score (Mean = 103.9 vs. 97.7, *p* ≤ .0001) than males. No sex differences were observed for motor development (*p* = .17). At 1 year of age, the mean and SD for the cognitive composite was 109.9 (SD *= *9.9), language composite was 107.7 (SD *= *13.1), and motor composite was 102.6 (SD *= *14.4), respectfully. Only performance on the language composite score at aged 1 year significantly differed, with females demonstrating a higher mean language score (Mean = 110.1 vs 105.5, *p* ≤ .0001) relative to males. There were positive correlations between cognitive scores (*r* = 0.14; *p* ≤ 0.001), and between language scores (*r* = 0.33, *p* ≤ 0.001) assessed at age 1 and 2 years.

**Table 1.** Covariates and their associations with infant neurodevelopment to 2 years of age (*n* = 405)

Outcome		BISD-III Cognitive Score		BISD-III Language Score		BISD-III Motor Score	
Variable	No.^a^ (%)	Mean (*SD*) or, *β* (95%CI)	*p-value*	Mean (*SD*)	*p-*value	Mean (*SD*)	*p*-value
*Categorical factors – Mean (SD)***^a^**							
**Gender**							
Female	199 (49.1)	108.4 (14.8)	≤0.001	103.9 (11.6)	≤0.001	99.7 (9.4)	0.17
Male	206 (50.9)	102.9 (13.2)		97.7 (11.3)		98.4 (9.7)	
**Maternal ethnicity**							
White	324 (80.6)	106.4 (14.2)	0.14	101.6 (11.4)	≤0.001	98.8 (9.4)	0.40
Asian	37 (9.2)	102.6 (14.9)		95.5 (13.0)^c^		98.7 (10.5)	
Other	41 (10.2)	103.1 (13.6)		99.4 (13.2)		101.0 (10.4)	
**Family income**							
≥ $60,000	376 (94.5)	106.0 (14.0)	0.25	102.3 (11.6)	0.22	99.3 (9.4)	0.32
< $60,000	22 (5.5)	102.5 (17.2)		98.0 (13.3)		97.2 (12.3)	
**Birth Mode**							
Vaginal, No IAP	208 (51.7)	107.4 (15.5)	0.04	101.6 (11.5)	0.05	99.8 (9.1)	0.29
Vaginal, IAP	88 (21.9)	104.1 (12.3)		101.6 (11.5)**^c^**		97.8 (9.6)	
Scheduled CS	49 (12.2)	101.3 (11.8)		96.9 (12.4)		97.7 (11.1)	
Emergence CS	57 (14.2)	105.2 (13.6)		99.1 (12.8)		99.2 (9.6)	
**Direct Antibiotic Exposure**							
Yes	72 (17.8)	104.4 (13.1)	0.20	99.8 (10.9)	0.44	99.2 (8.7)	0.92
No	333 (82.2)	105.9 (14.5)		100.9 (12.1)		99.0 (9.7)	
**Older Sibling**							
Yes	218 (53.8)	105.1 (13.8)		100.3 (12.0)		99.8 (9.8)	0.07
No	187 (46,2)	106.3 (14.7)	0.40	101.3 (11.7)	0.40	98.1 (9.2)	
**Ear Infection (0–12 months)**							
Yes	42 (11.4)	104.2 (12.8)	0.43	98.1 (9.5)	0.11	98.0 (9.3)	0.43
No	328 (88.7)	106.6 (14.5)		101.2 (12.2)		99.2 (9.3)	
**Breastfeeding, 6 months**							
None	107 (26.4)	103.8 (11.9)	0.25	98.6 (10.8)	0.03	98.9 (10.4)	0.95
Partial	229 (56.5)	106.0 (14.9)		100.8 (11.4)		99.1 (9.5)	
Exclusive	69 (17.1)	107.3 (15.0)		103.6 (14.2)**^c^**		99.4 (8.4)	
**Pre-pregnancy weight**							
Overweight	173 (44.4)	104.6 (13.8)	0.16	99.8 (11.5)	0.14	98.3 (10.2)	0.29
Normal weight	217 (56.6)	106.6 (14.7)		101.6 (12.1)		99.3 (9.0)	
*Continuous factors – β (95% CI)*^c^							
**Gestational age at delivery**	405 (100)	1.1 (0.1, 2.2)	0.03	1.2 (0.3, 2.1)	≤0.001	0.9 (0.2, 1.6)	0.01
**Maternal prenatal fruit intake**^d^	383 (94.6)	0.35 (−0.51, 1.2)	0.21	1.2 (0.5, 1.9)	≤0.001	−0.10 (−0.7, 0.5)	0.75
**Age at microbiota sampling, in months**	405 (100)	0.7 (−0.4, 1.8)	0.43	−0.07 (−0.9, 0.8)	0.88	0.22 (−0.5, 1.0)	0.55

Abbreviations: BISD-III = Bayley Infant Scales of Development Third Edition; SD = standard deviation; *β = Coefficient.*

*Note* Neurodevelopment was assessed in three domains: cognitive, language, and motor. The standardized population mean is 100 (standard deviation of 15). Higher scores indicate better abilities. Total sample (N) represents those participants with neurodevelopmental data collected at the 2-year study visit and 12-month microbiome sampling (mean = 12.5 months).

^a^Analyzed by *t*test or One-Way Analysis of Variance (ANOVA).

^b^Analyzed by linear regression.

^c^Significant group comparisons using Tukey post hoc test.

^d^Total fruit intake indicates the “5-a-day” method (calculated as sum of servings of fruit, not including juices, plus servings of juice per day).

### Identifying the infant gut microbiota clusters

#### 4-month microbiota clusters

**Figure 1.**
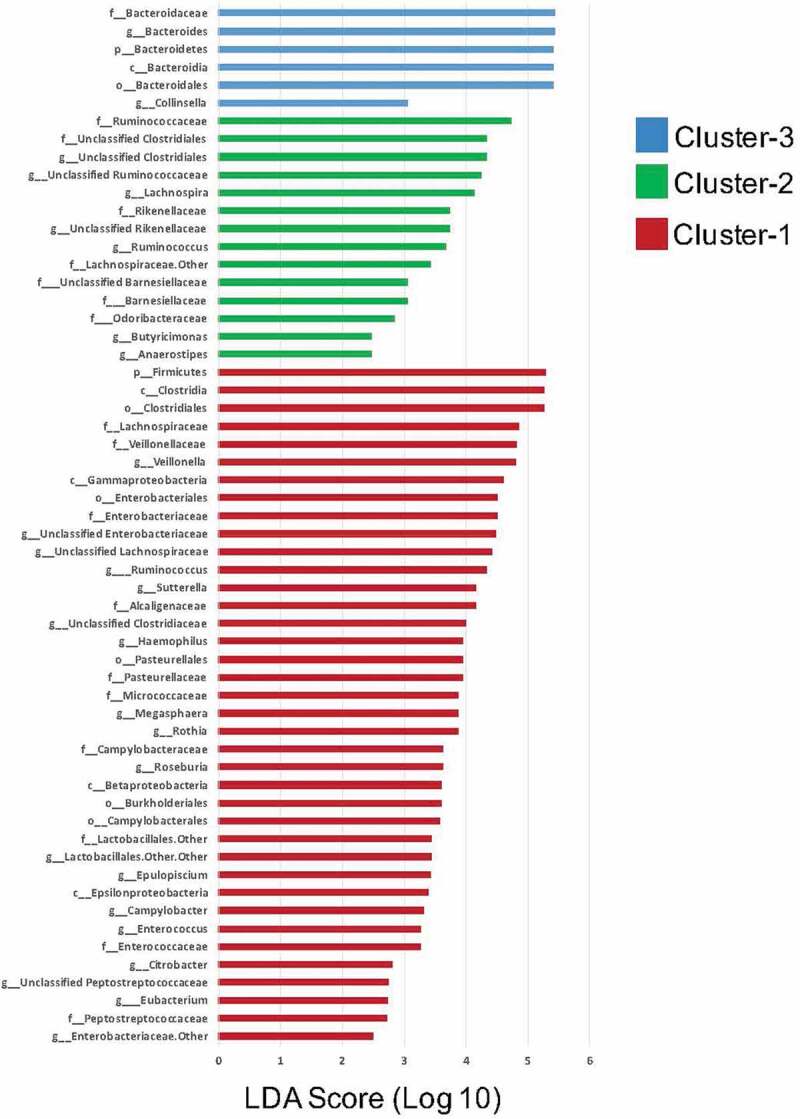
Linear discriminant analysis (LDA) scores for bacterial taxa differentially abundant in infant gut microbiota according to PAM Clusters at 12 months (LDA >2)

Separation of the bacteria populations by Partitioning around medoids (PAM) clustering identified three microbiota clusters at aged 4 months as follows (Figure 1): Proteobacteria and Firmicutes-dominant cluster (35.8%), Firmicutes-dominant cluster (24.7%) and Bacteroidetes-dominant cluster (39.5%). Since the 4-month clusters were not associated with BSID-III composite scores at 1 and 2 years of age they were not further characterized (presented later).

#### 12-month microbiota clusters

PAM clustering yielded three distinct, mutually-exclusive gut microbiota clusters shown in Figure S2. At 12 months, the LEfSe analysis (Figure S2) revealed a higher abundance of several Proteobacteria within Cluster 1 (22.4%, *n* = 91; Proteobacteria-dominant Cluster), a higher abundance of Firmicutes microbiota within Cluster 2 (46.0%, *n* = 186; Firmicutes-dominant Cluster), and a higher abundance of Bacteroidetes species within **Cluster 3** (31.6%, *n* = 128; Bacteroidetes-dominant Cluster).

The distribution of covariates across the three microbiota clusters are shown in Table 2.

**Table 2.** Distribution of infant and maternal characteristics according to each infant gut microbiota cluster group

	Microbiota at 1 year			
Descriptive Variable	Cluster 1 (*n* = 91/405) % (n)	Cluster 2 (*n* = 186/405) % (n)	Cluster 3 (*n* = 128/405) % (n)	*p-value*
*Categorical*				
Sex				
Male	47.3 (43/91)	55.9 (104/186)	46.1 (59/128)	0.17
Female	52.7 (48/91)	44.1 (82/186)	53.9 (69/128)	
Maternal ethnicity				
White	74.7 (68/91)	86.3 (158/186)	76.6 (98/128)	
Asian	16.5 (15/91)	3.8 (7/186)	11.7 (15/128)	
Other	8.8 (8/91)	9.8 (18/186)	11.7 (15/128)	
Family income				
High	95.5 (84/91)	92.3 (167/186)	88.7 (110/128)	0.71
Low	4.5 (4/91)	7.7 (14/186)	11.3 (14/128)	
Birth Mode				
Vaginal, NO IAP	42.9 (39/91)	54.3 (100/186)	54.3 (69/128)	≤0.001
Vaginal, IAP	22.0 (20/91)	17.9 (33/186)	27.6 (35/128)	
Scheduled CS	9.9 (9/91)	14.7 (27/186)	10.2 (13/128)	
Emergency CS	25.3 (23/91)	13.0 (24/186)	7.9 (10/128)	
Direct Antibiotic exposure 0–12 months				
Yes	19.8 (18/91)	16.1 (30/186)	18.8 (24/128)	0.71
No	80.2 (73)	83.9 (156)	81.3 (104)	
Older sibling				
Yes	37.4 (34)	67.2 (125)	45.7 (58)	≤0.001
No	62.6 (57)	32.8 (61)	54.3 (69)	
Ear infection (0–12 month)				
Yes	7.7 (7)	15.2 (28)	11.7 (15)	0.20
No	92.3 (84)	84.8 (156)	88.3 (113)	
Breastfeeding status at 6 months				
None	25.3 (23)	24.2 (45)	30.5 (39)	0.07
Partial	49.5 (45)	58.6 (109)	58.6 (75)	
Exclusive	25.3 (23)	17.2 (32)	10.9 (14)	
Maternal pre-pregnancy weight				
Overweight	38.6 (34)	45.6 (83)	46.7 (56)	0.46
Normal	61.4 (54)	54.4 (99)	53.3 (64)	
*Continuous*	Mean (SD)	Mean (SD)	Mean (SD)	
Gestational age at delivery	39.49 (1.37)	39.51 (1.27)	39.44 (1.38)	0.88
Age at microbiota sampling	12.21 (0.89)	12.67 (1.53)	12.33 (1.09)	≤0.001
Maternal prenatal fruit intake^a^	3.02 (1.71)	2.84 (1.49)	3.10 (1.86)	0.40

Abbreviations: PMCs = Proteobacteria; FMCs = Fimicutes; BMCs = Bacteroidetes; SD = standard deviation; IAP = intrapartum antibiotics; CS = cesarean section.

^a^Total fruit intake was defined as the “5-a-day” method calculated as sum of servings of fruit, not including juices, plus servings of juice per day.

Significantly more infants in Firmicutes-dominant Cluster 2 were born to a Caucasian mother relative to the other two cluster groups and they were likely to have an older sibling. Delivery by emergency cesarean and breastfeeding exclusivity was highest in Firmicutes-dominant Cluster 1. Other factors (antibiotic exposure, ear infection, maternal pre-pregnancy overweight, gestational age) known to influence infant gut microbiota did not differ among the three clusters. Notably, there were no sex differences by microbiota cluster. Next, we assessed the relationship between the same covariates and neurodevelopmental outcomes (cognitive, language, motor) shown in Table 1. Gender and birth mode were significantly associated with cognitive and language composite scores, while breastfeeding at 6-months, ethnicity, and maternal prenatal fruit intake were significantly associated with language compositive score (*p’s* <0.05). We found significant associations between gestational age and all three outcome measures; other more subtle differences in scores by study covariates are shown in Table 1.

## Infant microbiome and neurodevelopmental outcomes

### No associations between microbiota clusters in early infancy and neurodevelopmental outcomes

**Figure 2.**
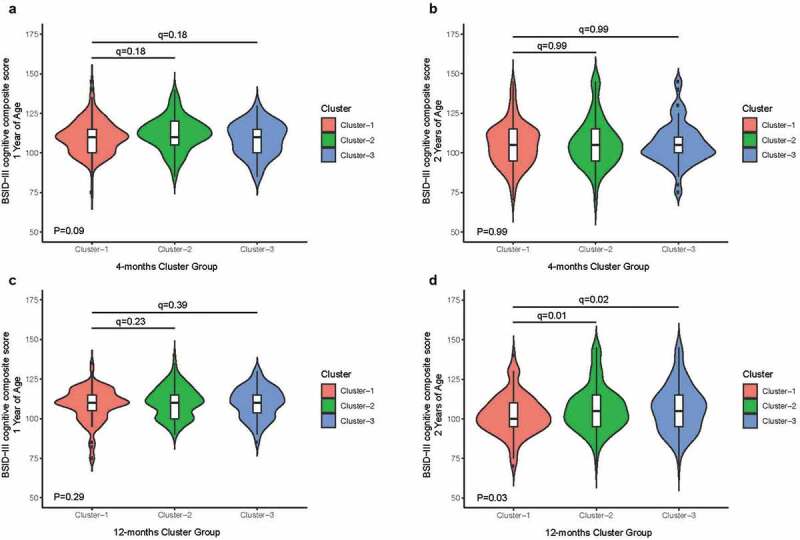
Associations between cognitive composite scores and microbiota cluster membership

We tested associations between the microbiota clusters analyzed at a mean age of 4 months with cognitive development (primary outcome). We found no associations between 4-month microbiota cluster membership and BSID-III cognitive composite scores at age 1 year (*q* = 0.18) and age 2 years (*q* = 0.99, Figure 2a and b) in univariate analysis. Similarly, there were no other significant associations found between the 4-month microbiota clusters membership and the other BSID-III language and motor composite scores (not presented).

### Evidence of associations between microbiota clusters in late infancy and neurodevelopmental outcomes

#### Microbial diversity and richness differ among 12-month clusters

Total microbial species richness and diversity of infant gut microbiota differed among the three clusters (all FDRp’s<0.001; Table S2). In brief, Proteobacteria-dominant Cluster 1 had the lowest total species richness and diversity, but highest richness within the Proteobacteria phylum. Firmicutes-dominant Cluster 2 had the highest total species richness and diversity, especially within the Fimicutes phylum. In **Bacteroidetes-dominant Cluster 3**, richness within the Bacteroidetes phylum was highest without observed differences in total phylogenetic diversity.

#### Bacteroidetes and Firmicutes microbiota clusters in late infancy are associated with enhanced neurodevelopmental outcomes at age 2 in all infants

We assessed the relationship between microbiota clusters analyzed at a mean age of 12 months and the primary outcome cognitive development using the Proteobacteria-dominant cluster as the reference group. We found no significant associations between the 12-month cluster membership and concurrent BSID-III cognitive composite scores at 1 year of age in multivariate analysis (Table S3 and Figure 2c). Next, we tested the relationship between 12-month microbiota clusters and the BSID-II cognitive composite score at 2 years of age. Relative to the Proteobacteria-dominant Cluster 1, infants in microbiota Bacteroidetes-dominant Cluster 3 and Firmicutes-dominant Cluster 2 exhibited higher scores for cognitive development at 2 years indicating better abilities (4.8 points, 95%CI: 0.8–8.7, FDRp = .02; and 5.3 points, 95%CI: 1.4–9.1, FDRp = .01, respectively; Figure 2d and Table 3).

**Table 3.** Crude and adjusted effects of microbiota at 12 months and neurodevelopment to 2 years of age

Microbiota Cluster Group	BSID-III composite scores at 2 years of age (*n* = 405)													
	Cognitive				Language				Motor					
	Crude		Adjusted^a^		Crude		Adjusted^a^		Crude			Adjusted^a^		
	Beta (95%CI)	q-value	Beta (95%CI)	q-value	Beta (95%CI)	q-value	Beta (95%CI)	q-value	Beta (95%CI)	q-value		Beta (95%CI)	q-value	
**Cluster 1**	*Reference*				*Reference*				*Reference*					
**Cluster 2**	4.0 (0.5, 7.5)	0.03	5.3 (1.4, 9.1)	0.01	2.2 (−0.8, 5.2)	0.14	4.1 (1.1, 7.2)	≤0.001	2.6 (0.2, 4.9)	0.04		3.0 (0.4, 5.6)	0.03	
**Cluster 3**	4.2 (0.4, 8.0)	0.03	4.8 (0.8, 8.7)	0.02	3.2 (0.1, 6.4)	0.10	4.2 (1.1, 7.4)	≤0.001	2.8 (0.3, 5.4)	0.04		3.1 (0.4, 5.9)	0.03	
	**BSID-III composite scores 1–2 years (*n* = 401)**													
**Cluster 1**	*Reference*				*Reference*				*Reference*					
**Cluster 2**	3.2 (0.8, 5.7)	0.02	3.8 (1.3, 6.4)	≤0.001	1.9 (−0.7, 4.5)	0.15	2.8 (0.3, 5.4)	0.03	2.4 (0.0, 4.9)		0.10	2.0 (−0.4, 4.5)		0.14
**Cluster 3**	3.2 (0.4, 5.6)	0.03	3.4 (0.8, 6.0)	0.02	2.8 (0.0, 5.6)	0.10	3.2 (0.5, 5.8)	0.03	1.8 (−0.7, 4.4)		0.17	2.0 (−0.6, 4.5)		0.14

Abbreviations: BSID-III: Bayley Infant Scales of Development Third Edition.

*Note* Analyzed by separate Generalized Linear Models and separate Linear Mixed Models with adjustments for the same covariates. The standardized population mean is 100 (standard deviation of 15). Higher scores indicate better abilities.

^a^Covariates include birth mode, sex, maternal ethnicity, older sibling, breastfeeding status at 6 months, family income, maternal overweight, and age at sampling.

We further determined independence of associations between the 12-month microbiota clusters and the BSID-III language and motor composite scores shown in Table 3. Here, we found that the Bacteroidetes-dominant and Firmicutes-dominant microbiota clusters were independently associated with higher scores indicating better language and motor abilities at age 2. Relative to the Proteobacteria-dominant Cluster 1, the **Bacteroidetes-dominant Cluster 3** group had a 4.2-point increase in language composite score (94%CI: 1.1, 7.4, FDRp≤0.001), while infants in Firmicutes-dominant Cluster 2 showed a 4.1-point increase in language composite score (95%CI: 1.1, 7.2 FDRp≤0.001) in fully adjusted models. Similarly, infants in Firmicutes-dominant and Bacteroidetes-dominant microbiota clusters also had significantly higher scores relative to the Proteobacteria-dominant Cluster 1 in the motor domain of the BSID-III in adjusted analyses (all FDRp’s<0.05; shown in Table 3). These associations were independent of family income, maternal ethnicity, birth mode, breastfeeding status, direct antibiotic exposure, older sibling, gestational fruit intake, maternal overweight, and age at sampling.

We then tested associations between the microbiome cluster group membership and change in performance on the BSID-III scales from 1 to 2 years using linear mixed model (LMM) analysis and Proteobacteria-dominant cluster as the reference group. After adjustment for confounders, we found that the Bacteroidetes-dominant and Firmicutes-dominant microbiota clusters were independently and positively associated with cognitive and language performance change between 1 and 2 years old shown in Table 3. **Bacteroidetes-dominant Cluster 3** was associated with a 3.4-point (95%CI: 0.8, 6.0, FDR*p* = .02) increase in cognitive performance between 1 and 2 years. **Bacteroidetes-dominant Cluster 3** group was also associated with a 3.2-point (95%CI: 0.5, 5.8, FDR*p* = .03) increase in language performance but not for motor performance (FDR*p*>.05; shown in Table 3). Similarly, Firmicutes-dominant Cluster 2 group was positively associated with an increase in cognitive (3.8-points; 95%CI: 1.3, 6.4, FDR*p*≤0.001) and language performance (2.8-points; 95%CI: 0.3, 5.4, FDR*p* = .03) from 1 to 2 years but not for motor performance (FDR*p>*.05; shown in Table 3).

#### Sex specific associations between the Bacteroidetes microbiota cluster in late infancy and neurodevelopmental outcomes

Next, we ascertained whether associations observed between microbiota cluster membership and neurodevelopmental domains occurred in a sex-specific manner. In a stratified analysis and relative to the Proteobacteria-dominant Cluster 1, male infants within **Bacteroidetes-dominant Cluster 3** exhibited a 5.9-point increase to cognitive development score at age 2 (95%CI: 0.6, 11.1, FDRp = .06; Table 4). **Bacteroidetes-dominant Cluster 3** was also associated with enhanced language development in males at this age (7.9 points; 95%CI: 3.4, 12.3, FDR *p* ≤ 0.001; Table 4). Male infants in the Firmicutes-dominant Cluster 2 group scored higher on language development (5.1 points; 95%CI: 0.9, 9.3, FDR*p* = .02; Table 4) but not cognitive development at age 2 (FDRp>.05; Table 4). These associations remained statistically significant after adjustment for several covariates including birth mode, maternal prenatal fruit intake, maternal overweight, and breastfeeding status. However, we found that microbiota cluster type was unrelated to motor development among male infants.

**Table 4.** Crude and adjusted effects of microbiota at 12 months and infant neurodevelopment, stratified by sex

Microbiota Cluster Group	BSID-III composite scores at 2 years of age (*n* = 405) by sex											
	Cognitive				Language				Motor			
	Crude		Adjusted		Crude		Adjusted		Crude		Adjusted	
	Beta (95%CI)	q-value	Beta (95%CI)	q-value	Beta (95%CI)	q-value	Beta (95%CI)	q-value	Beta (95%CI)	q-value	Beta (95%CI)	q-value
**Cluster 1**	*Reference*				*Reference*				*Reference*			
**Cluster 2** Females	4.8 (−0.5,10.0)	0.15	5.0 (−0.8,10.8)	0.18	2.6 (−1.5, 6.7)	0.44	2.6 (−1.6, 6.8)	0.46	4.3 (1.0, 7.5)	0.02	3.9 (0.2, 7.7)	0.08
Males	4.3 (−0.3, 8.9)	0.07	4.8 (−0.13, 9.8)	0.16	3.2 (−0.7, 7.1)	0.11	5.1 (0.9, 9.3)	0.02	1.1 (−2.4, 4.5)	0.55	1.5 (−2.2, 5.1)	0.42
**Cluster 3** Females	3.4 (−2.0, 8.8)	0.22	3.4 (−2.4, 9.2)	0.25	0.5 (−3.8, 4.7)	0.83	0.3 (−3.9, 4.6)	0.88	4.1 (0.7, 7.5)	0.02	3.3 (−0.5, 7.1)	0.09
Males	5.0 (−0.1, 10.1)	0.07	5.9 (0.6, 11.1)	0.06	6.2 (1.9, 10.6)	0.01	7.9 (3.4, 12.3)	≤0.001	1.3 (−2.5, 5.1)	0.55	2.8 (−1.1, 6.7)	0.34
	**BSID-III composite scores 1–2 years (*n* = 401) by sex**											
**Cluster 1**	*Reference*				*Reference*				*Reference*			
**Cluster 2** Females	3.5 (0.0, 7.0)	0.10	3.2 (−0.05, 6.8)	0.20	1.4 (−2.1, 4.9)	0.86	0.8 (−2.8,4.3)	0.99	3.3 (0.0, 6.7)	0.06	2.1 (−1.4, 5.6)	0.52
Males	3.9 (0.5, 7.2)	0.02	4.1 (0.6, 7.6)	0.03	4.0 (0.5, 7.6)	0.03	4.7 (1.0, 8.3)	0.02	2.0 (−1.5, 5.4)	0.26	1.8 (−1.8, 5.3)	0.35
**Cluster 3** Females	1.4 (−2.3, 5.0)	0.46	1.4 (−2.2, 5.1)	0.46	0.3 (−3.4, 3.9)	0.89	0.0 (−3.6, 3.5)	0.99	1.1 (−2.4, 4.7)	0.53	1.2 (−2.3, 4.7)	0.52
Males	4.9 (1.3, 8.6)	0.02	4.9 (1.1, 8.7)	0.03	6.1 (2.2, 9.9)	≤0.001	6.2 (2.3, 10.1)	≤0.001	2.6 (−1.1, 6.3)	0.26	2.8 (−1.1, 6.6)	0.34

Among female infants, both Firmicutes-dominant and Bacteroidetes-dominant microbiota clusters were significantly associated with enhanced motor development at age 2, but these associations did not survive FDR correction following adjustment for covariates. We also observed no significant associations among females between the microbiota clusters and cognitive or language development.

In a stratified LMM analysis, male infants in the **Bacteroidetes-dominant Cluster 3** showed an increase in cognitive composite score (4.9-point; 95%CI: 1.1, 8.7, FDR*p* = .03; Table 4) and language composite score (6.2-point; 95%CI: 2.3, 10.1, FDR≤0.001; Table 4) from 1 to 2 years, but not females. Similarly, male infants classified into the Firmicutes-dominant group showed an increase in cognitive composite score (4.1-point; 95%CI: 0.6, 7.6, FDR*p* = .03) and language composite score (4.7-point; 95%CI: 1.0, 8.3, FDR*p* = .02) from 1 to 2 years, but not females. No sex-dependent differences were observed for motor performance from 1 to 2 years (Table 4).

#### Correlation between abundances of microbiota keystone species at age 1 year and neurodevelopmental outcomes

Based on GLM models, different species of *Bacteroides* including *B. fragilis, B. uniformis*, and unclassified *Bacteroides* were positively associated with increased cognitive development (all FDRp’s<0.05). Similarly, *B. uniformis* and unclassified *Bacteroides* were associated with improved language development, while unclassified Prevotella were positively associated with motor development (all FDRp’s<0.05). Shown in Tables S4, S5, and S6.

#### Microbial interaction networks associated with 12-month clusters

**Figure 3.**
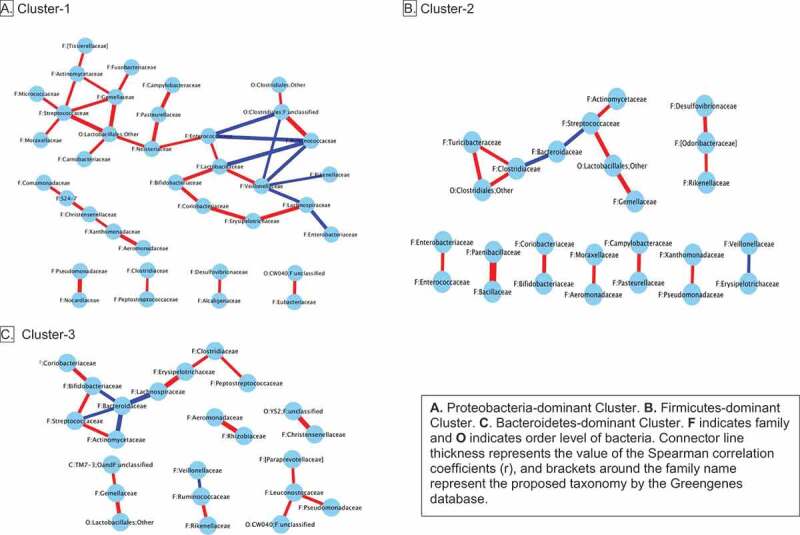
Microbiota interaction networks for microbiota clusters at 12 months

A total of 37 microbial families were involved in the co-occurrence network of Proteobacteria-dominant Cluster 1, while this number was 25 and 23 families in Firmicutes-dominant Cluster 2 and Bacteroidetes-dominant Cluster 3, respectively. A unique feature of the microbial network of Cluster 1 was the lack of Bacterioidaceae’s involvement. However, the abundance of Bacterioidaceae was inversely associated with that of Streptococcaceae in the other two clusters. Shown in Figure 3a–c.

#### Microbiota metabolic function differs among 12-month clusters

**Figure 4.**
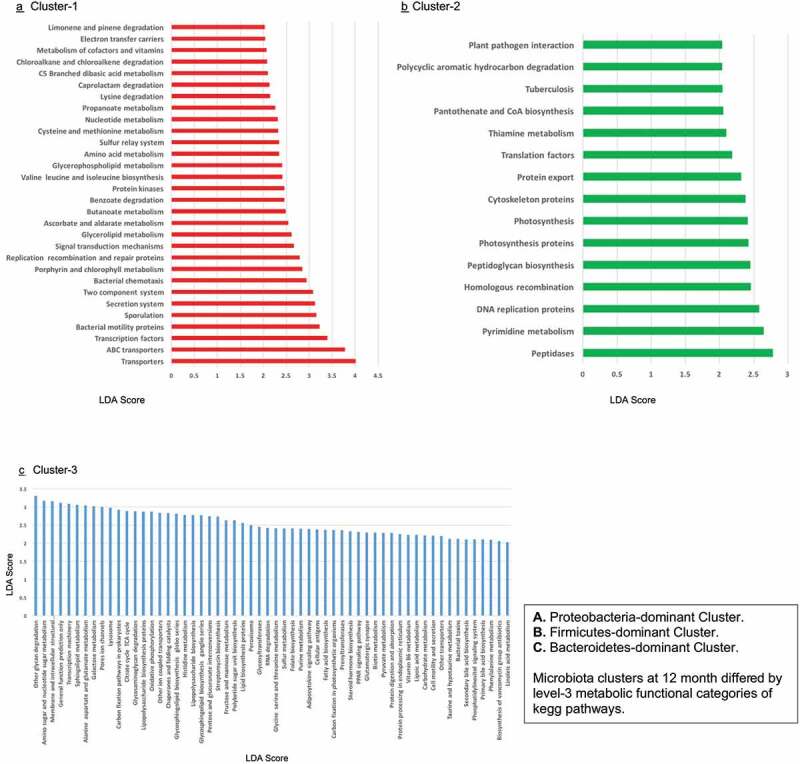
Microbiota metabolic function at 12 months according to cluster group membership

Microbiota clusters differed by level-3 metabolic functional categories of KEGG pathways (Figure 4a–c). Among the three clusters, **Bacteroidetes-dominant Cluster 3** was enriched with multiple metabolic functions including sphingolipid metabolism and glycosphingolipid biosynthesis. Moreover, genes involved in metabolism of folate, biotin, pyruvate, vitamin B6, lipoic acid and fatty acid biosynthesis were enriched in this cluster.

## Discussion

In this general population birth cohort of 405 infants, a beneficial impact of gut microbiota during infancy was documented for early neurodevelopmental outcomes. We observed that infants with an enriched abundance of *Bacteroides* in their gut microbiota at 1 year had more favorable BSID-III cognitive and language development at 2 years. In fact, infants with a Bacteroidetes-dominant microbial composition achieved 4.8-point and 4.2-point higher cognitive and language development scores, respectively, which represents close to half a standard deviation difference in performance. These observed effect sizes, and especially those in boys (7.9-point higher score for language) are similar to ones found for breastfeeding^33^ and infant sleep duration.^34^ Depletion of *Bacteroides* in gut microbiota is also characteristic of children diagnosed with autism spectrum disorder at age 2–3 years.^35^ Moreover, our findings were driven by male infants since statistical significance was not found for Bacteroidetes-dominant gut microbiota and advanced cognitive or language development in female infants. The gut-brain axis of boys seems to be more susceptible to disruptions in the gut microbiome,^36^ which has been linked to alterations to the brain’s serotonergic system in germ-free animal models.^37^ Hence, our study contributes to growing evidence that neurodevelopmental outcomes are shaped by the gut microbial composition of infants in a sex-dependent manner. Importantly, we found statistical significance with the Bacteroidetes cluster in male infants for change to BSID-III cognition and language scores between 1 and 2 years of age, adding stronger evidence for a causal association.

Bacteroidetes species may play a key role in promoting neurodevelopment during a critical time in late infancy when myelination and expanded connectivity of neuronal networks normally occur; when these processes are slowed, pervasive developmental delay can result.^38,39^ With successive increases to gut microbial species richness in both young and older infants, greater connectivity in brain areas that support cognitive development and language acquisition has been reported.^40,41^ We observed that presence of the Bacteroidetes-cluster (with the highest species richness of Bacteroidetes), as well as higher relative abundance of genus *Bacteroides* at 1 year, were associated with improved neurodevelopmental outcomes. Our results are quite similar to the 12-month *Bacteroides* association with neurodevelopmental outcomes at age 2 in the Carlson et al.^27^ study, and they are consistent with critical periods of postnatal brain development. Their study^27^ also identified *Bacteroides*-dominance to be characteristic of 1 years olds with large gray-matter volumes in the superior occipital gyrus, the back region of the brain also shown to have reduced integration with other brain networks in preschool children with autism versus controls.^42^ Contrary to the VDAART study^28^ (Ages and Stages questionnaire), we did not observe associations between 4-month microbial composition and neurodevelopmental outcomes in infancy. Our findings are supported by the microbiota gut-brain-axis concept, which suggests that the presence of the gut microbiota plays a key role in the gut-brain-axis and in neurodevelopment. This is evident in germ-free mice models whereby the absence of a gut microbiota causes alterations in cognition and memory,^43^ and social development, a core deficit in autism.^44^ Murine models of autism point to the potential role of *Bacteroides fragilis* in ameliorating defects in communicative and sensorimotor behaviors, potentially through a modulatory effect on serum metabolites, including sphingolipids.^11^

Our findings also have biological plausibility in terms of the actions of microbial short-chain fatty acid (SCFA) metabolite pathways on the developing brain in the infant. The SCFA propionate, is chiefly produced by the Bacteroidetes species which become more prominent in the gut of later infancy.^45–47^ A tendency for lower fecal propionate levels has been reported in school children with pervasive developmental and language disorders.^48^ Through its conversion to odd-chain fatty acids such as pentadecanoic acid, propionate has been posited to play a role in ganglioside production and myelination of neurons.^49–51^ Indeed, we found sphingolipid metabolism, especially the biosynthesis of glycosphingolipid (ganglio series), to be enriched in the Bacteroidetes-dominant cluster. The absence of ganglio-series gangliosides (sialylated glycosphingolipids) in mutant mice leads to early developmental deficits in reflexes, strength, coordination, and balance,^52^ as well as to progressive motor and sensory dysfunction, and deterioration in spatial learning and memory.^52,53^ In humans, the expression of gangliosides undergoes significant change during the development of the brain, largely attributed to the normal functioning and maintenance of the brain.^49^ When sphingomyelin levels are higher, be it in infant serum or supplemented formula, or through breastfeeding, preschool children perform better on neurocognitive scales and exhibit improved myelination of the brain.^54,55^ Further, similar to the study by Carlson et al.^27^ we also observed greater abundance of functional genes related to the production of vitamins and cofactors such biotin, lipoic acid, folate and vitamin B6 in the cluster dominated by Bacteroidetes.

Gut microbiome pathways to neurodevelopment may also operate through the interactions of genus *Bacteroides* with other gut microbiota. Multiple associations between the abundance of microbial families were apparent within the Bacteroidetes-dominant microbiota cluster, including an inverse correlation between the abundance of Bacteroidaceae and Streptococcaceae. Group B Streptococcus (GBS: *S. agalactiae*) is a leading cause of newborn sepsis and meningitis, and the main indication for maternal intrapartum prophylaxis during vaginal delivery.^56^ A recent meta-analysis^57^ confirmed neurodevelopmental impairment to develop in 20% of infants with neonatal GBS infection. Others too have found higher propionate levels, the main metabolite produced by Bacteroidetes species, with lower abundance of *Streptococcus* in term infants.^58^ Unable to compete with Bacteroidetes and other microbes in an increasing anaerobic environment, *Streptococcus* abundance declines to very low levels in gut microbiota as infants grow older; yet, when Bacteroidetes are depleted in the gut following emergency cesarean or maternal intrapartum prophylaxis, some streptococcal species continue to thrive in older infants who had been exclusively breastfed for 3–4 months.^46^ In our study, 28% of infants in Bacteroidetes-dominant microbiota cluster were born following maternal intrapartum prophylaxis for GBS, 18% had been delivered by cesarean and 70% had been breastfed for 6-months. A short duration of breastfeeding may explain why the streptococcal interaction was not found with the *Bacteroides* cluster in the Carlson et al. study,^27^ where half of the infants in this cluster had received formula by age 1.

## Study strengths and limitations

There are several strengths of our study: i) high-throughput deep sequencing to profile gut microbiota at two critical periods of microbiota over the first year of life in relation to brain development between 1 and 2 years, ii) objective assessment of neurodevelopment by experts using a well-validated and widely-used gold standard measure, and iii) a large sample size that enabled adjustment for ethnicity and early life covariates. An important limitation of this work is that the PICRUSt analysis of function can only infer potential mechanisms since it predicts metagenomic function according to the 16S sequences of reference genomes. Future studies should also consider a shotgun metagenomic approach to examine microbial function with more depth to further assess causality and mechanisms. We were also unable to examine infants’ high risk for neurodevelopmental morbidity as the CHILD Cohort Study excluded preterm birth below 35 weeks. Other high-risk groups excluded from our study were families of low socioeconomic status. Further studies are required to investigate the generalizability of our findings to other populations.

## Conclusions

In a general population, we found evidence for the influence of gut microbiota, namely the *Bacteroides* species, and associated sphingolipid synthesis in late infancy on subsequent neurodevelopmental outcomes. Our study suggests a greater effect size among male infants, particularly for cognitive and language abilities. Future studies are needed to confirm these findings and examine the impact of the infant gut microbiome on more complex tasks at later neurodevelopmental stages.

## Methods

### Study design and population

This was a microbiome study of 577 infants with neurodevelopmental outcomes at ages 1 and 2 years old, as part of a substudy at the Edmonton site of the CHILD Cohort Study.^59,60^ This sample included a subset of infants with fecal samples collected at approximately 4 months (from 414 infants) and/or 1 year follow-up (from 405 infants). Enrollment methods for their expectant mothers in the general population and their predominantly term newborns (35+ weeks gestation) have been described in detail elsewhere (www.childstudy.ca).^59^ Mothers of studied infants were enrolled during pregnancy between January 2009 and December 2012. Their infants were seen at a planned 3–4 months, 1 year, and 2 years study visits.^59,60^ Informed consent was obtained from all mothers and the study was approved by the Human Research Ethics Board at the University of Alberta (Pro00002099).

### Neurodevelopmental assessments

Infant neurodevelopmental assessments, using the Bayley Scale of Infant Development Third Edition (BSID-III),^61^ were completed at 1 year and 2 years of age, during the day at a time when parents felt their infant was most alert (i.e. not during a scheduled naptime). The BSID-III is a validated objective measure of cognitive, language, motor development for infants aged 1 to 42 months. The BSID-III cognitive scale (91-items) assesses visual preference, attention, memory, exploration, manipulation, and concept formation; the language scale assesses receptive communication (49-items) and expressive communication (48-items); and the motor scale assess gross motor (72-items) and fine motor (66-items) skills. It’s cognitive (0.91), language (0.93), and motor (0.92) subscales have high reliability coefficients, and good test-retest stability with coefficients around 0.80.^61,62^ A registered educational psychologist trained research staff to administer the BSID-III instrument and conducted semiannual assessments. Testing of participants was completed during a single session by two research staff. All scores were obtained based on the child’s chronological age at the time of testing. Raw scores were converted to scaled scores, then to composite scores. The standardized population mean for the composite score is 100 (standard deviation of 15). A higher score on the BSID-III scales indicates better abilities.

### Confounding variables

Data from study questionnaires or hospital birth records were obtained to create covariates as follows: infant sex, maternal ethnicity (Caucasian, Asian or other), family income (<, ≥60,000 USD), maternal pre-pregnancy overweight, birth mode (vaginal, elective or emergency cesarean section), maternal intrapartum antibiotic prophylaxis (IAP), any infant oral antibiotic treatment until age 1, any infant ear infections, breastfeeding status (exclusively, partially, or not breastfeed) and older siblingship. Maternal prenatal fruit intake (“5-a-day” method) the sum of “servings of fruit, not including juices, “plus servings of juice” per day,^63^ which we previously found to be associated with infant cognition,^64^ was based on the 5-day method from a modified 174-item, self-reported Food Frequency Questionnaire.^63^

### Fecal microbiota analysis

Gut microbiota were profiled by 16S rRNA gene sequencing in fecal samples collected from 414 infants at a mean age of 4.2 months (SD = 1.24) and from 405 infants at a mean age of 12.5 months (SD = 1.29) during planned study visits. Within the funding scope of the CHILD Cohort Study, these collection points strategically represented a sample during the peak of breastfeeding in Canadian infants and a post-weaning sample at the end of infancy. Sample collection, DNA extraction and amplification methods have been previously described in detail^21,65^ (see online supplementary content for details).

### Statistical analysis

Study sample characteristics were compared to those of infants missing neurodevelopment and microbiome data using Chi-square or ANOVA tests. OTU relative abundances were summarized at the phylum, family and genus levels of taxonomy with QIIME software. Microbial alpha-diversity was calculated with four standard indices (Chao1, Shannon, Simpson, and Faith Phylogenetic Diversity).

(i) ***Clustering analysis***

To identify microbiota clusters according to genus abundance, all samples were clustered using the partitioning around medoids (PAM) clustering algorithm, described by Arumugan et al.^66^ and tested in infants;^67^ the optimal number of clusters was determined by the Calinski-Harabasz index and Silhouette width. The linear discriminant analysis effect size (LEfSE) with an LDA log cutoff of 2 was applied to identify unique taxa that differentiated the microbiota cluster groups and could be used to name clusters by dominant microbiota. Thereafter, clusters were characterized and compared according to microbial diversity (non-parametric Kruskal-Wallis and post hoc Dunn tests with a false discovery rate (FDR) correction), co-occurrence taxon abundance networks and PICRUSt metabolic function.^68^

(i) ***General linear modeling (GLM)***

Our units of analysis were cognitive (primary outcome), and language and motor (secondary outcomes) BSID-III composite scores. Univariate analysis (t-test, ANOVA, Pearson correlations) identified covariates that differed (*p* < .05) among microbiota clusters and BSID-III scores. Using GLM, associations between microbiota clusters with cluster group 1 as the reference and BSID-III composite scores at aged 1 and 2 were tested separately in fully adjusted models. Adjusted for covariates, associations between BSID-III composite scores and taxon relative abundance were determined by GLM.

(i) ***Linear mixed model (LMM)***

To explore the influence of microbiome group membership on neurodevelopmental scores, we used linear effects models with BSID-III composite scores from the repeated aged 1 and aged 2 visit as outcomes. Sampling age, neurodevelopmental assessment visit age, and covariates were entered into the model as random terms. Statistical significance was defined as a two-sided q-value <0.05, after FDR correction of the *p*-value for multiple comparison. GLM and LMM analyses were conducted in SAS Software version 9.4 (SAS).

## Supplementary Material

Supplemental Material

## Data Availability

The data that supports the findings of this study are available from the corresponding author and CHILD Cohort Study coordinators upon reasonable request. These data, including study data, are securely stored in the https://childdb.ca database.
