# Supplementary material for: Bacteroides-dominant gut microbiome of late infancy is associated with enhanced neurodevelopment
Source: Gut Microbes. 2021 Jun 16;13(1):1930875. doi: 10.1080/19490976.2021.1930875 (PMC8210878; doi:10.1080/19490976.2021.1930875)
Supplement: Supplemental Material [file KGMI_A_1930875_SM9588.docx]

**Supplementary Online Content**

**Appendix.** Detailed Methods.

**Figure S1.** Linear Discriminant Analysis (LDA) Effect Size (LEfSe) Plot for 4-month Microbiota Clusters.

**Figure S2.** Cluster Analysis of Infant Gut Microbiota at 12 months.

**Table S1**. Characteristics of Infants with Bayley Scales of Infant Development (BSID-III) Assessments at Age 1 and 2 Years, With or Without Microbiota Data (*n*=573).

**Table S2**. Relative Abundance of Dominant Phyla and Families in Fecal Microbiota of Infants at aged 12.5 months.

**Table S3**. Crude and Adjusted Effects of Microbiota Sampled at 12.5 months and Cognitive Development at Aged 1 year.

**Table S4**. Relative Abundance of Dominant Phyla and Families in Fecal Microbiota of Infants at aged 12.5 month, According to the Mean composite Score of Age 2 years Cognitive Development.

**Table S5**. Relative Abundance of Dominant Phyla and Families in Fecal Microbiota of Infants at aged 12.5 months, According to the Mean Composite Score of Age 2 years Language Development.

**Table S6**. Relative Abundance of Dominant Phyla and Families in Fecal Microbiota of Infants at aged 12.5 months, According to the Mean Composite Score of Age 2 years Motor Development.

**eAppendix.** Detailed Methods

***Fecal microbiota analysis***

Stool samples were collected on 405 infants using a standardized protocol during a planned 3-4 months and 1 year study visit. Mothers were provided with Tegaderm liners for their infant’s diaper at least 3 days prior to the visit. Fecal samples (fresh or refrigerated for a short period) were aliquoted using a stainless steel depyrogenated spactula. Samples were immediately refrigerated during transport and stored at -80’C until analysis. DNA extraction and amplification were conducted using whole genome DNA. Whole genome DNA was extracted from 80 to 200 mg of fecal samples using the QIamp DNA stool mini kit (Qiagen, Venlo, the Netherlands). The Bacterial 16S rRNA gene, hypervariable region V4, was amplified by PCR using universal bacterial primers: V4-515f 5′ AAT GAT ACG GCG ACC ACC GAG ATC TAC ACT ATG GTA ATT GTG TGC CAG CMG CCG CGG TAA-3′, V4-806r:5′–CAA GCA GAA GAC GGC ATA CGA GAT XXXXXXXXXXXX AGT CAG TCA GCC GGA CTA CHV GGG TWT CTA AT-3′. The reverse primer was barcoded so that each sample could be uniquely identified post-sequencing (denoted in the primer sequence by Xs). Each PCR mixture (25’l) contained 12.5’l 2l Kapa2G Hotstart mix (Kapa Biosystems, Wilmington, MA), molecular biology reagent grade water (Sigma-Aldrich, ST. Louis, MO, USA), 0.6lM primer, and 2’l bacterial template DNA (5ng/’l). PCR consusted of an initial DNA denaturation step (94’C, 3 min) followed by 20 cycles of denaturation (94’C, 30 sec), annealing (50’C, 30 sec) and elongation (72’C, 30 sec), performed on a OTC-200 Thermal Cycler (MJ Research, ST. Bruno, QC, Canada). Reactions performed in triplicate and pooled with a negative control included in each run. 100 ng of product was condensed using an Amicon’ Ultra-4 30K centrifugal filter (Millipore, Billerica, MA, USA), run through a 1.4% agarose gel extracted, and cleaned with the GENECLEAN’ Turbo Kit (MP Biomedicals Inc., Solon, OH, USA).

**Sequencing** **and bioinformatics**

Pooled PCR amplicons subjected to paired-end sequencing by Illumina MiSeq. QIIME pipeline (v 1.6.0, Quantitative Insights into Microbial Ecology, qime.org): forward and reverse reads assembled (PandaSeq) for a final length of 144bp. Unassembled sequences discarded. Demultiplexed and filtered against the Greengenes bacterial reference database (v 12.10) to remove all sequences with less than 60% similarity. Sequences clustered with Usearch61 at 97% sequence similarity against the Greengenes database (closed-picking algorithm). Taxonomic assignment was achieved using the RDP classifier constrained by Greengenes. Operational taxonomic units (OTUs) with overall relative abundance below 0.0001 excluded from subsequent analyses. Total of 110 million reads restrained (median 3.1x105 per sample, range 8.1x104 - 1.0x106), representing 1127 unique OTUs. Data rarefied to 80, 000 sequences per sample for analyses. Microbiota composition at 1 year of age was characterized using high-throughput 16S rRNA sequencing.

**Figure S1.** Linear Discriminant Analysis (LDA) Effect Size (LEfSe) Plot for 4-month Microbiota Clusters.


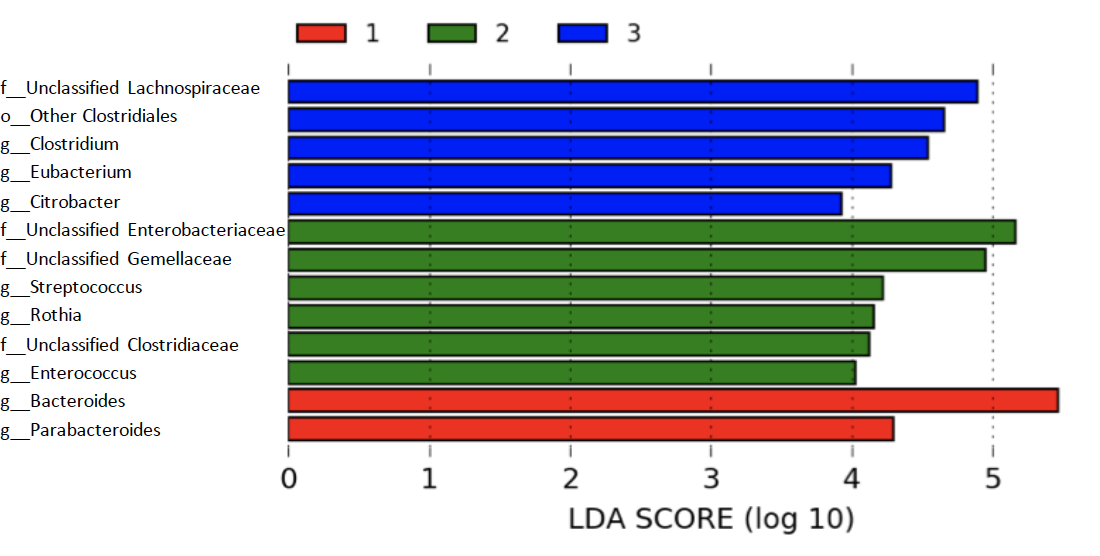


**Legend**: Linear Discriminant Analysis (LDA) scores provided for differential taxon abundance between **Cluster 1** – Proteobacteria- and Firmicutes-dominant cluster-group, **Cluster 2** - Firmicutes-dominant cluster-group, and **Cluster 3** - Bacteroidetes-dominant cluster-group.

**Figure S2:** Cluster Analysis of Infant Gut Microbiota at 12 months.

1a. 1b.

**** **
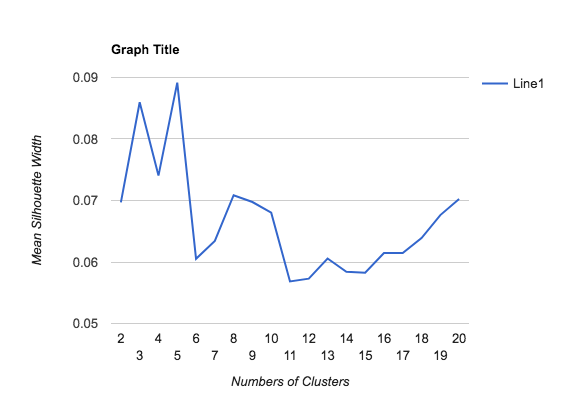
**

1c.

**Proteobacteria**

**Firmicutes**

**Bacteroidetes**

*Note* 1a. and 1b. Partitioning around medoids (PAM) clustering algorithm; 1c. Represents the three microbiota-dominant cluster groups.

| **Table S1.** Characteristics of Infants with Bayley Scales of Infant Development Assessments at Age 1 and 2 Years, With or Without Microbiota Data (*n*=577). | | | |
| --- | --- | --- | --- |
| Descriptive variable | BSID-III data present without  microbiota data  (*n*=172) | BSID-III data present with microbiota data  (*n*=405)^ab^ |  |
| *Categorical* | % (*n*) | % (*n*) | *p-value* |
| Sex: Male | 53.5 (92/172) | 50.9 (206/405) | 0.56 |
| Maternal ethnicity: White | 73.3 (126/172) | 80.6 (324/405) | 0.20 |
| Family income: > $60,000 | 84.9 (146/172) | 94.5 (376/405) | 0.01 |
| Maternal education: postsecondary or higher | 88.7 (141/172) | 91.1 (369/405) | 0.01 |
| Older sibling: yes | 62.8 (108/172) | 53.8 (218/405) | 0.05 |
| C-section delivery: yes^b^ | 21.5 (37/172) | 26.4 (106/405) | 0.42 |
| Breastfed, 6-months: yes^c^ | 70.9 (78/172) | 73.6 (298/405) | 0.87 |
| *Continuous* | Mean (SD) | Mean (SD) |  |
| Gestational age | 39.5 (1.4) | 39.5 (1.3) | 0.99 |
| Maternal fruit intake during pregnancy^d^ | 2.7 (1.9) | 3.0 (1.7) | 0.21 |
| Age at 4-month microbiota sampling in months | - | 4.2 (1.2) | - |
| Age at 12-month microbiota sampling in months | - | 12.5 (1.3) | - |

Analysed by chi-squared statistical test

Abbreviations: BSID-III: Bayley Infant Scales of Development Third Edition.

^a^ Only four study participants had neurodevelopmental outcome data at one assessment and microbiome data.

^b^ No study participants with fecal samples were missing neurodevelopment data at 1 or 2 years old.

^b^ Birth mode via c-section includes total for planned and emergency delivery.

^b^ Breastfeeding status includes partial or exclusively breastfed at 6-months.

^d^ Total fruit intake based on the “5-a-day” method calculated as the sum of servings of fruit, not including juices, plus servings of juice per day.

**Table S2**. Relative Abundance of Dominant Phyla and Families in Fecal Microbiota of Infants at aged 12.5 months

| **Taxa level** | **Alpha-diversity indices** | **Cluster 1**  **Median** | **Cluster 2**  **Median** | **Cluster 3**  **Median** | **q-value** |
| --- | --- | --- | --- | --- | --- |
| **Overall** | Faith’s Phylogenetic diversity | 14.8 | 17.6 | 14.4 | <0.001 |
|  | Chao1 | 293.4 | 377.7 | 314.8 | <0.001 |
|  | Observed species | 212.0 | 269.0 | 218.0 | <0.001 |
|  | Shannon | 4.3 | 4.5 | 3.9 | <0.001 |
|  | Simpson | 0.9 | 0.9 | 0.8 | <0.001 |
| **Actinobacteria** | Faith’s Phylogenetic diversity | 1.7 | 1.7 | 1.6 | 0.01 |
|  | Chao1 | 18.0 | 15.6 | 15.0 | 0.02 |
|  | Observed species | 15.0 | 14.0 | 13.0 | <0.001 |
|  | Shannon | 1.6 | 1.7 | 1.6 | 0.01 |
|  | Simpson | 0.5 | 0.5 | 0.5 | 0.01 |
| **Bacteroidetes** | Faith’s Phylogenetic diversity | 2.0 | 3.3 | 3.3 | <0.001 |
|  | Chao1 | 22.0 | 89.1 | 92.8 | <0.001 |
|  | Observed species | 11.0 | 66.5 | 70.0 | <0.001 |
|  | Shannon | 2.2 | 2.3 | 2.1 | 0.14 |
|  | Simpson | 0.7 | 0.6 | 0.6 | 0.39 |
| **Fimicutes** | Faith’s Phylogenetic diversity | 8.7 | 10.9 | 7.8 | <0.001 |
|  | Chao1 | 188.3 | 235.7 | 169.0 | <0.001 |
|  | Observed species | 138.0 | 164.5 | 104.5 | <0.001 |
|  | Shannon | 4.0 | 4.6 | 4.1 | <0.001 |
|  | Simpson | 0.9 | 0.9 | 1.0 | <0.001 |
| **Proteobacteria** | Faith’s Phylogenetic diversity | 2.3 | 1.8 | 1.9 | <0.001 |
|  | Chao1 | 54.2 | 25.9 | 35.1 | <0.001 |
|  | Observed species | 33.0 | 15.0 | 19.0 | <0.001 |
|  | Shannon | 1.8 | 1.7 | 1.8 | 0.25 |
|  | Simpson | 0.5 | 0.5 | 0.5 | 0.93 |

Note: Analyzed by Kruskal–Wallis one-way analysis of variance.

| **Table S3**. Crude and Adjusted Effects of Microbiota Sampled at 12.5 months and Cognitive Development at Aged 1 year. | | | | | | | | | | | | |
| --- | --- | --- | --- | --- | --- | --- | --- | --- | --- | --- | --- | --- |
| **Microbiota Cluster Group** | **BSID-III composite scores at 1 year of age (*n*=405)** | | | | | | | | | | | |
|  | **Cognitive** | | | | **Language** | | | | **Motor** | | | |
|  | **Crude** | | **Adjusted** | | **Crude** | | **Adjusted** | | **Crude** | | **Adjusted** | |
|  | Beta  (95%CI) | q-value | Beta  (95%CI) | q-value | Beta  (95%CI) | q-value | Beta  (95%CI) | q-value | Beta (95%CI) | q-value | Beta (95%CI) | q-value |
| Cluster 1 | *Reference* | | | | *Reference* | | | | *Reference* | | | |
| Cluster 2 | 1.9  (-0.6, 4.4) | 0.28 | 2.2  (-0.5, 5.0) | 0.22 | 2.2  (-1.1, 5.5) | 0.21 | 3.4  (-0.2, 7.0) | 0.06 | 1.8  (-1.9, 5.4) | 0.68 | 0.5  (-3.5, 4.5) | 0.80 |
| Cluster 3 | 1.2  (-1.4, 3.9) | 0.36 | 1.5  (-1.3, 4.3) | 0.30 | 2.3  (-1.3, 5.8) | 0.21 | 3.7  (-0.003, -7.4) | 0.06 | 0.3  (-3.6, 4.2) | 0.89 | 1.4  (-2.5, 5.2) | 0.80 |

| Abbreviations: BSID-III: Bayley Infant Scales of Development Third Edition.  *Note* Analyzed by separate generalized linear models with adjustments for the same covariates. The standardized population mean is 100 (standard deviation of 15). Higher scores indicate better abilities. | ^a^ Covariates include birth mode, sex, maternal ethnicity, older sibling, breastfeeding status at 6 months, family income, maternal pregnancy fruit intake (5-a-day method sum of servings of fruit, not including juices, plus servings of juice per day), maternal overweight, and age at sampling. |
| --- | --- |

| **Table S4**. Relative Abundance of Dominant Phyla and Families in Fecal Microbiota of Infants at aged 12.5 months, According to the Mean composite Score of Age 2 Years Cognitive Development. | | | | | |
| --- | --- | --- | --- | --- | --- |
| **Present *vs.* Absent (Y/N)**  **OTU#** | **Taxonomy** | **BSID-III cognitive composite score at 2 years** | | | |
|  |  | **Beta** | **SE** | **95% CI** | ***p*-value** |
| **4411138** | k__Bacteria; p__Actinobacteria; c__Actinobacteria; o__Actinomycetales; f__Micrococcaceae; g__Rothia; s__mucilaginosa | -4.65 | 1.78 | -8.14, -1.16 | ≤0.001 |
| **3486634** | k__Bacteria; p__Bacteroidetes; c__Bacteroidia; o__Bacteroidales; f__Bacteroidaceae; g__Bacteroides; s__uniformis | 3.98 | 1.62 | 0.80, 7.16 | 0.01 |
| **4250634** | k__Bacteria; p__Bacteroidetes; c__Bacteroidia; o__Bacteroidales; f__Bacteroidaceae; g__Bacteroides; s__uniformis | 3.53 | 1.66 | 0.29, 6.78 | 0.03 |
| **2243510** | k__Bacteria; p__Bacteroidetes; c__Bacteroidia; o__Bacteroidales; f__Bacteroidaceae; g__Bacteroides; s__ | 3.37 | 1.58 | 0.27, 6.47 | 0.03 |
| **344154** | k__Bacteria; p__Bacteroidetes; c__Bacteroidia; o__Bacteroidales; f__Bacteroidaceae; g__Bacteroides; s__uniformis | 3.23 | 1.56 | 0.18, 6.28 | 0.04 |
| **4278525** | k__Bacteria; p__Bacteroidetes; c__Bacteroidia; o__Bacteroidales; f__Bacteroidaceae; g__Bacteroides; s__uniformis | 3.25 | 1.47 | 0.36, 6.14 | 0.03 |
| **4477861** | k__Bacteria; p__Bacteroidetes; c__Bacteroidia; o__Bacteroidales; f__Bacteroidaceae; g__Bacteroides; s__ | 3.98 | 1.51 | 1.03, 6.94 | ≤0.001 |
| **4437814** | k__Bacteria; p__Bacteroidetes; c__Bacteroidia; o__Bacteroidales; f__Bacteroidaceae; g__Bacteroides; s__uniformis | 4.10 | 1.57 | 1.02, 7.19 | ≤0.001 |
| **190309** | k__Bacteria; p__Bacteroidetes; c__Bacteroidia; o__Bacteroidales; f__Bacteroidaceae; g__Bacteroides; s__uniformis | 1.71 | 1.55 | -1.33, 4.75 | 0.27 |
| **175535** | k__Bacteria; p__Bacteroidetes; c__Bacteroidia; o__Bacteroidales; f__Bacteroidaceae; g__Bacteroides; s__ | 2.11 | 1.51 | -0.84, 5.06 | 0.16 |
| **4457872** | k__Bacteria; p__Bacteroidetes; c__Bacteroidia; o__Bacteroidales; f__Bacteroidaceae; g__Bacteroides; s__fragilis | 3.53 | 1.62 | 0.36, 6.70 | 0.03 |
| **328472** | k__Bacteria; p__Actinobacteria; c__Actinobacteria; o__Actinomycetales; f__Actinomycetaceae; g__Varibaculum; s__ | -7.16 | 3.38 | -13.8, -0.53 | 0.03 |
| **4352747** | k__Bacteria; p__Bacteroidetes; c__Bacteroidia; o__Bacteroidales; f__Bacteroidaceae; g__Bacteroides; s__fragilis | 5.87 | 2.17 | 1.62, 10.12 | ≤0.001 |

| Abbreviations: BSID-III: Bayley Infant Scales of Development Third Edition. |
| --- |
| *Note* Analyzed by Generalized Linear Model adjusted for covariates. |
| Covariates include birth mode, gender, maternal race, birth order (siblings), breastfeeding status at 6 months, direct antibiotic exposure (0-12) months, family income, maternal pregnancy fruit intake (5-a-day method sum of servings of fruit, not including juices, plus servings of juice per day), and age at microbiota sampling. |

| **Table S5**. Relative Abundance of Dominant Phyla and Families in Fecal Microbiota of Infants at aged 12.5 months, According to the Mean Composite Score of Age 2 years Language Development. | | | | | |
| --- | --- | --- | --- | --- | --- |
| **Present *vs.* Absent (Y/N)**  **OTU#** | **Taxonomy** | **BSID-III Language composite score at 2 years** | | | |
|  |  | **Beta** | **SE** | **95% CI** | ***p*-value** |
| **4411138** | k__Bacteria; p__Actinobacteria; c__Actinobacteria; o__Actinomycetales; f__Micrococcaceae; g__Rothia; s__mucilaginosa | -4.79 | 1.40 | -7.54, -2.04 | ≤0.001 |
| **4383052** | k__Bacteria; p__Actinobacteria; c__Actinobacteria; o__Actinomycetales; f__Actinomycetaceae; g__Actinomyces; s__ | -2.44 | 1.17 | -4.74, -0.14 | 0.04 |
| **4477861** | k__Bacteria; p__Bacteroidetes; c__Bacteroidia; o__Bacteroidales; f__Bacteroidaceae; g__Bacteroides; s__ | 4.85 | 1.17 | 2.56, 7.15 | ≤0.001 |
| **4301298** | k__Bacteria; p__Bacteroidetes; c__Bacteroidia; o__Bacteroidales; f__Bacteroidaceae; g__Bacteroides; s__ | 7.40 | 4.24 | -0.92, 15.72 | 0.08 |
| **4250634** | k__Bacteria; p__Bacteroidetes; c__Bacteroidia; o__Bacteroidales; f__Bacteroidaceae; g__Bacteroides; s__uniformis | 3.68 | 1.31 | 1.12, 6.24 | 0.01 |
| **4278525** | k__Bacteria; p__Bacteroidetes; c__Bacteroidia; o__Bacteroidales; f__Bacteroidaceae; g__Bacteroides; s__ | 2.84 | 1.17 | 0.55, 5.12 | 0.02 |
| **3486634** | k__Bacteria; p__Bacteroidetes; c__Bacteroidia; o__Bacteroidales; f__Bacteroidaceae; g__Bacteroides; s__ | 2.92 | 1.29 | 0.40, 5.44 | 0.02 |
| **175535** | k__Bacteria; p__Bacteroidetes; c__Bacteroidia; o__Bacteroidales; f__Bacteroidaceae; g__Bacteroides; s__ | 2.13 | 1.19 | -0.20, 4.46 | 0.07 |

| Abbreviations: BSID-III: Bayley Infant Scales of Development Third Edition. |
| --- |
| *Note* Analyzed by Generalized Linear Model adjusted for covariates. |
| Covariates include birth mode, gender, maternal race, birth order (siblings), breastfeeding status at 6 months, direct antibiotic exposure (0-12) months, family income, maternal pregnancy fruit intake (5-a-day method sum of servings of fruit, not including juices, plus servings of juice per day), and age at microbiota sampling. |

| **Table S6**. Relative Abundance of Dominant Phyla and Families in Fecal Microbiota of Infants at aged 12.5 months, According to the Mean Composite Score of Age 2 Years Motor Development. | | | | | |
| --- | --- | --- | --- | --- | --- |
| **Present *vs.* Absent (Y/N)**  **OTU#** | **Taxonomy** | **BSID-III motor composite score at 2 years** | | | |
|  |  | **Beta** | **SE** | **95% CI** | ***p*-value** |
| 4383052 | k__Bacteria; p__Actinobacteria; c__Actinobacteria; o__Actinomycetales; f__Actinomycetaceae; g__Actinomyces; s__ | -3.23 | 1.00 | -5.18, -1.27 | ≤0.001 |
| 12574 | k__Bacteria; p__Proteobacteria; c__Betaproteobacteria; o__Methylophilales; f__Methylophilaceae; g__; s__ | -5.56 | 1.70 | -8.89, -2.24 | ≤0.001 |
| 4474759 | k__Bacteria; p__Bacteroidetes; c__Bacteroidia; o__Bacteroidales; f__[Paraprevotellaceae]; g__[Prevotella]; s__ | 11.94 | 5.52 | 1.12, 22.8 | 0.03 |
| 4336070 | k__Bacteria; p__Actinobacteria; c__Actinobacteria; o__Actinomycetales; f__Actinomycetaceae; g__Actinomyces; s__ | -2.44 | 1.24 | -4.87, -0.01 | 0.05 |
| 332968 | k__Bacteria; p__Bacteroidetes; c__Bacteroidia; o__Bacteroidales; f__[Paraprevotellaceae]; g__[Prevotella]; s__ | 16.16 | 6.82 | 2.79, 29.5 | 0.02 |
| 526682 | k__Bacteria; p__Actinobacteria; c__Actinobacteria; o__Actinomycetales; f__Actinomycetaceae; g__Actinomyces; s__ | -1.67 | 1.25 | -4.12, 0.79 | 0.18 |
| 4343627 | k__Bacteria; p__Bacteroidetes; c__Bacteroidia; o__Bacteroidales; f__Bacteroidaceae; g__Bacteroides; s__fragilis | 1.11 | 1.01 | -0.88, 3.10 | 0.27 |
| 130663 | k__Bacteria; p__Bacteroidetes; c__Bacteroidia; o__Bacteroidales; f__Bacteroidaceae; g__Bacteroides; s__fragilis | 2.24 | 1.20 | -0.11, 4.59 | 0.06 |

| Abbreviations: BSID-III: Bayley Infant Scales of Development Third Edition. |
| --- |
| *Note* Analyzed by Generalized Linear Model adjusted for covariates. |
| Covariates include birth mode, gender, maternal race, birth order (siblings), breastfeeding status at 6 months, direct antibiotic exposure (0-12) months, family income, maternal pregnancy fruit intake (5-a-day method sum of servings of fruit, not including juices, plus servings of juice per day), and age at microbiota sampling. |
